# Factors Associated With the Use of Over-the-Counter Sleep Aids Among Jazan University Students

**DOI:** 10.7759/cureus.67447

**Published:** 2024-08-21

**Authors:** Moaddey Alfarhan, Muath Haqwi, Abdulrahman H Musayyikh, Ibrahim J Alhiqwi, Ibrahim A Maadi, Majed M Faqeeh, Layla A Wushayli, Mashael M Sawadi, Faisal Y Tawhari, Ahmed M Hodani

**Affiliations:** 1 Department of Clinical Practice, College of Pharmacy, Jazan University, Jizan, SAU; 2 College of Pharmacy, Jazan University, Jizan, SAU; 3 College of Medicine, Jazan University, Jizan, SAU

**Keywords:** saudi arabia, jazan university, over-the-counter medications, sleep aids, insomnia

## Abstract

Background: Sleep aids, classified by their mechanisms of action, can promote sleep but may be misused, leading to harm. Exercise and pharmacological interventions like antihistamines, melatonin, and benzodiazepines also help manage sleep disorders. In Saudi Arabia, sleep disorders are prevalent, especially among university students.

Objectives: Our study examines the prevalence and usage of the medication containing diphenhydramine hydrochloride, among Jazan University students, aiming to inform better practices and highlight related risks and benefits.

Methods: A cross-sectional design study was conducted among Jazan University students in Saudi Arabia. The sampling of data utilized random selection. Data was cleaned in Excel and analyzed using IBM SPSS Statistics for Windows, Version 29 (Released 2023; IBM Corp., Armonk, New York, United States).

Results: Our study comprised 437 participants from Jazan University aged 18-25 years. The majority reported earning less than 5000 SAR monthly and were unmarried (91.8%). Remarkably, 13.7% of participants were diagnosed with insomnia. Sleep aid containing diphenhydramine hydrochloride, utilized for mild to moderate pain relief and sleep induction, was the most prevalent medication, with 56.3% of participants having used it and 9.4% using it continuously for over 10 nights. Significant predictors for usage of sleep aids containing diphenhydramine hydrochloride included gender (p=0.041), with male students exhibiting higher usage rates, and college type (p<0.001), particularly medical students. Multivariate analysis confirmed male gender and enrollment in medical colleges as robust predictors. Age, income, marital status, and employment variables showed no significant associations.

Conclusions: Our study highlights a considerable prevalence of sleep aids containing diphenhydramine hydrochloride usage among Jazan University students, notably influenced by gender and college type. Male gender and enrollment in medical colleges emerged as significant predictors of their usage.

## Introduction

Sleep aids, encompassing medications and herbal remedies, are designed to facilitate sleep through various mechanisms. Some aim to align with the body’s natural circadian rhythms, while others work by inhibiting brain neurotransmitters responsible for wakefulness. Notably, improper use of sleep aids can result in adverse effects or diminished therapeutic outcomes [1]. Misuse includes exceeding recommended dosages, prolonged use beyond prescription guidelines, or unsupervised consumption, all of which can lead to negative consequences [2]. In contrast to pharmacological solutions, exercise has been identified as a beneficial sleep promoter. Epidemiological and experimental research highlights that moderate aerobic activities like walking or cycling can enhance both sleep quality and duration by regulating the internal clock and increasing endorphin levels [3]. However, when medication is necessary, options include antihistamines such as diphenhydramine and chlorpheniramine, natural substances like melatonin and 5-hydroxytryptophan, and prescription medications like benzodiazepines. These should be administered with medical oversight to mitigate risks of side effects and dependency [4].

The global prevalence of sleep disorders stands at 27.3% [5]. Still, a study done by Aldhafiri and his colleagues indicates a much higher rate of 61.6% in Saudi Arabia, underscoring the significant impact of these conditions [6]. Sleep-related issues are notably common in higher education settings and affect many students. About 7% of students in such institutions resort to medications to manage their sleep problems [4]. These issues are not rare, with around 30% of university students experiencing sleep difficulties, which one-third find particularly distressing or challenging [7,8]. The link between poor sleep and mental health concerns like anxiety and depression underscores the critical need to address sleep disorders within this demographic. Yet, these disorders may not receive the attention they deserve, often dismissed as a mere aspect of student life [7,8].

This underestimation of sleep disorders has led to a study aimed at evaluating the prevalence and usage of sleep aids, including sleep aids containing diphenhydramine hydrochloride, among adults in Jazan, Saudi Arabia. The study aimed to understand why sleep aid containing diphenhydramine hydrochloride is misused and quantify its usage. The findings will enhance existing knowledge about sleep aid use, particularly sleep aids containing diphenhydramine hydrochloride, in Jazan. Insights gained could illuminate the extent of the issue, potential concerns surrounding misuse, and the effectiveness of sleep aid containing diphenhydramine hydrochloride in improving sleep quality. Such information is valuable for healthcare providers, policymakers, and individuals regarding the proper use of sleep aids and their associated benefits or risks. Additionally, the study seeks to identify factors linked to the use and misuse of over-the-counter (OTC) sleep aids among students at Jazan University.

## Materials and methods

Study design

This descriptive cross-sectional study was conducted using a self-administered online survey at Jazan University, Saudi Arabia. The research ethics committee at Jazan University approved the study. The participants were adult students aged 18 years or older who agreed to participate in the study. The exclusion criteria included the following: not being a student of Jazan University or those who refuse to participate in the study

Sample size

The study comprised 437 participants, meeting the predetermined sample size requirement based on statistical calculations for a 95% confidence level and a margin of error of 0.05. Participants encompassed both males and females, selected through random sampling from the student body of Jazan University to ensure comprehensive representation. A total of 440 participants were initially included, and after excluding 3 due to incompletion or illegibility, the net outcome was 437, meeting the sample size requirement. The sample size was determined to be 382 participants. Raosoft, Inc.'s sample size calculator was used for the calculation.

Data collection tool

‏The validated questionnaire, obtained from previous research [9], was divided into four sections. Section 1 covered demographics and personal information, section 2 evaluated sleep problems, section 3 assessed knowledge about OTC sleep aids, and section 4 measured sleep aids containing diphenhydramine hydrochloride usage. The survey was conducted via an electronic survey between 13 April and 19 May 2024.

Data analysis

A comprehensive statistical analysis was performed on the dataset, which included both descriptive and inferential methodologies. Initially, a descriptive analysis was conducted to summarize the participants’ demographic characteristics, such as age, gender, and other features, providing an overview of the study population. Subsequently, inferential analyses and the chi-square test were employed to examine the association between OTC usage and different features. Additionally, binary logistic regression was used to identify the adjusted predictors of OTC usage of sleep aids containing diphenhydramine hydrochloride. Statistical significance was established at a p-value of 0.05 or lower and a 95% Confidence Interval. All statistical analyses were performed using IBM SPSS Statistics for Windows, Version 29 (Released 2023; IBM Corp., Armonk, New York, United States).

Ethical consideration

Ethical approval for this study was obtained from the Research Ethics Committee of Jazan University, Saudi Arabia, with protocol approval number (REC-45/09/1016). All participants provided informed consent, and their confidentiality and privacy were strictly maintained by anonymizing personal identifiers in the dataset. Participation in the study was voluntary, and participants could withdraw at any time without consequences.

## Results

Our study included 437 participants from Jazan University who were assessed for OTC sleep medication use (Table 1). There is a slight male majority (n=246, 56.3%) compared to females (n=191, 43.7%). The predominant age group was 18-25 years (n=385, 88.1%), followed by those under 18 years (n=35, 8.0%) and those over 25 years (n=17, 3.9%). Most participants reported a monthly income of less than 5000 SAR (n=351, 80.3%), while smaller proportions earned between 5000 and 10000 SAR (n=49, 11.2%) and more than 10,000 SAR (n=37, 8.5%). The vast majority were single (n=401, 91.8%), with only a few being married or divorced (n=36, 8.2%). The sample was almost evenly split between non-medical (n=219, 50.1%) and medical college students (n=218, 49.9%). Additionally, most participants were not employed (n=414, 94.7%), with a minor segment being employed (n=23, 5.3%).

**Table 1:** Baseline characteristics of the study participants

Demographic Variables		n (%)
Gender	Female	191 (43.7)
	Male	246 (56.3)
Age (years)	<18	35 (8.0)
	18-25	385 (88.1)
	>25	17 (3.9)
Monthly income (SAR)	<5000	351 (80.3)
	5000-10000	49 (11.2)
	>10,000	37 (8.5)
Marital status	Single	401 (91.8)
	Married/Divorced	36 (8.2)
Which college	Non-Medical	219 (50.1)
	Medical	218 (49.9)
Employment status	No	414 (94.7)
	Yes	23 (5.3)

In our survey, we inquired about the participants' sleep quality. Notably, 60 (13.7%) reported being diagnosed with insomnia by a doctor, while the majority did not (Table 2). Over the past month, 93 (21.3%) students never experienced sleep trouble due to pain, while 113 (25.9%) reported this scarcely, 143 (32.7%) sometimes, 46 (10.5%) mostly, and 42 (9.6%) always (Table 2). When assessing overall sleep quality in the past month, 89 (20.4%) rated it as bad, 154 (35.2%) as good, 132 (30.2%) as very good, and 62 (14.2%) as excellent (Table 2).

**Table 2:** Sleep problems among Jazan University students

Sleep Quality and Insomnia Assessment		n (%)
Have you ever been diagnosed with insomnia by a doctor?	No	377 (86.3)
	Yes	60 (13.7)
During the past month, how many times have you had trouble sleeping because you are in pain?	Never	93 (21.3)
	Scarcely	113 (25.9)
	Sometimes	143 (32.7)
	Mostly	46 (10.5)
	Always	42 (9.6)
Over the past month, how would you rate your overall sleep quality?	Bad	89 (20.4)
	Good	154 (35.2)
	Very Good	132 (30.2)
	Excellent	62 (14.2)

**Figure 1:**
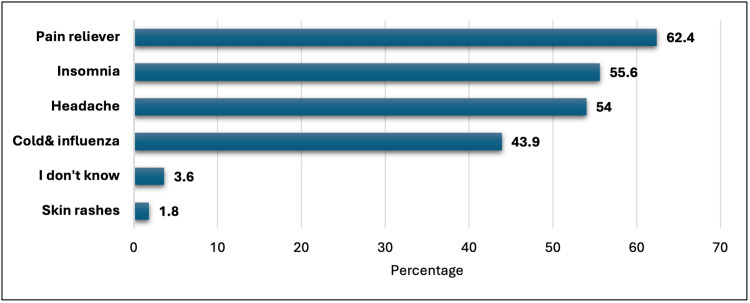
Causes of using over-the-counter sleep aids among Jazan University students

The results showed that the majority of students (62.4%) used OTC sleep aids as a pain reliever at night, while 55.6% of students reported using them for insomnia (Figure 1). Additionally, 54% used sleep aids for headaches, and 43.9% used them for cold and influenza. A smaller percentage of students reported using sleep aids for reasons such as skin rashes (1.8%) and not knowing why they were using them (3.6%).

In order to study the knowledge of sleep aid medications, we asked the students about different sleep aids, including medication containing diphenhydramine hydrochloride, chlorpheniramine maleate, melatonin, valerian, and St. John’s Wort. Among the 437 students surveyed, 387 (88.5%) were familiar with diphenhydramine hydrochloride as a sleep aid medication (Table 3). Additionally, 184 students (42.1%) were familiar with chlorpheniramine maleate, 145 students (33.1%) with melatonin, 105 students (24.0%) with valerian, and 91 students (20.8%) with St. John's Wort.

**Table 3:** Knowledge about sleep aid medications

Sleep Aids Medications	n (%)
Diphenhydramine hydrochloride	387 (88.5)
Chlorpheniramine maleate	184 (42.1)
Melatonin	145 (33.1)
Valerian	105 (24.0)
St. John's Wort	91 (20.8)

Since the diphenhydramine-containing sleep aid was the most commonly known sleep aid among the participants, we asked the students further questions to determine their knowledge about diphenhydramine-containing medication and its safety. We found that 193 respondents (44.2%) were neutral, 136 (31.1%) agreed it was safe, 55 (12.6%) strongly agreed, 41 (9.4%) disagreed, and 12 (2.7%) strongly disagreed (Table 4). Regarding the perception of treating chronic insomnia with medication containing diphenhydramine hydrochloride, 227 (51.9%) students responded "Maybe," 173 (39.6%) said "No," and 37 (8.5%) believed it could be effective (Table 4). Our results indicate that more than half of the students (n=246, 56.3%) had used medication containing diphenhydramine hydrochloride to help with sleep, although only 61 (14.0%) were currently using it (Table 4). We asked the students if they had used a sleep aid containing diphenhydramine hydrochloride recently, and we found that 76 (17.4%) had used it in the past 30 days (Table 4). Diphenhydramine hydrochloride usage peaked during specific times of the year for 127 (29.1%) students and 41 (9.4%) students reported continuous usage for more than 10 nights in a row (Table 4). The majority of participants who used medication containing diphenhydramine hydrochloride were influenced by family or friends (n=114, 26.1%), while 73 (16.7%) were advised by medical personnel, and 46 (10.5%) were influenced by the internet or social media (Table 4).

**Table 4:** Knowledge about diphenhydramine hydrochloride usage-related features

Diphenhydramine Hydrochloride Usage and Experience Survey		n (%)
Do you think it is safe to use medication containing diphenhydramine hydrochloride?	Strongly Disagree	12 (2.7)
	Disagree	41 (9.4)
	Neutral	193 (44.2)
	Agree	136 (31.1)
	Strongly Agree	55 (12.6)
Do you think you can successfully treat chronic insomnia with diphenhydramine hydrochloride-containing medication?	No	173 (39.6)
	Maybe	227 (51.9)
	Yes	37 (8.5)
Have you ever used a medication that contains diphenhydramine hydrochloride to help you sleep?	No	191 (43.7)
	Yes	246 (56.3)
If yes, are you currently using a medication containing diphenhydramine hydrochloride to help you sleep?	No	376 (86.0)
	Yes	61 (14.0)
Have you used products containing diphenhydramine hydrochloride to help you sleep in the past 30 days?	No	361 (82.6)
	Yes	76 (17.4)
Do you use diphenhydramine hydrochloride-containing medication at a specific time of the year?	No	310 (70.9)
	Yes	127 (29.1)
Have you taken a medication containing diphenhydramine hydrochloride more than 10 nights in a row a month?	No	396 (90.6)
	Yes	41 (9.4)
I used a medication containing diphenhydramine hydrochloride recommended by:	I don’t use it	192 (43.9)
	Family/Friends	114 (26.1)
	Medical Personal	73 (16.7)
	Internet/social media	46 (10.5)
	Personal Decision	12 (2.7)
Have you ever experienced any side effects when taking diphenhydramine hydrochloride-containing medication for sleep?	No	379 (86.7)
	Yes	58 (13.3)

**Figure 2:**
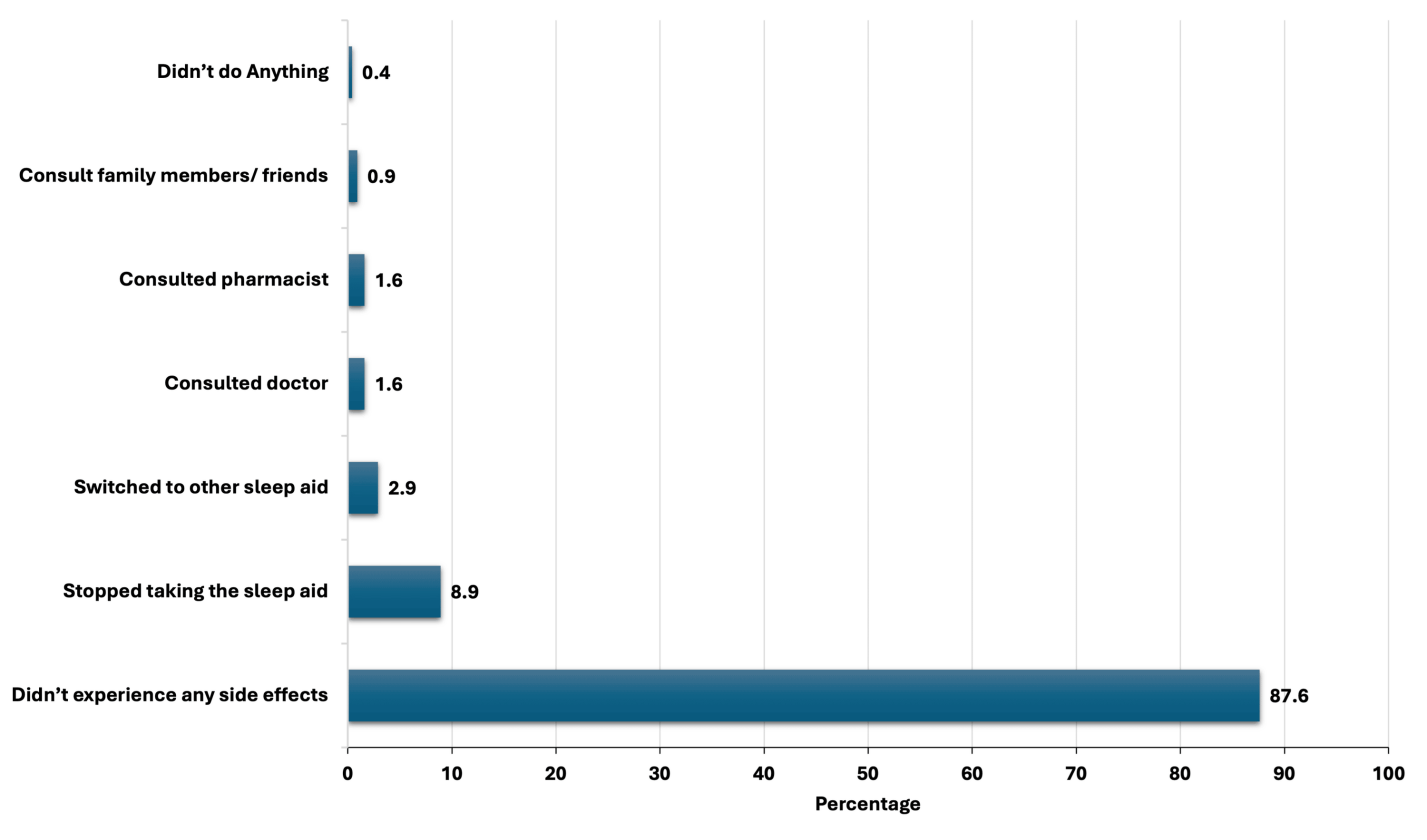
Methods of side effect management of over-the-counter sleep medications

A significant majority, 383 students (87.6%), reported not experiencing any side effects (Figure 2). Among those who did, various methods were employed to manage these effects. Stopping the use of the sleep aids was the most common method, with 39 students (8.9%) choosing this approach (Figure 2). Notably, 13 (2.9%) switched to another sleep aid (Figure 2). Consulting a doctor and consulting a pharmacist were each reported by seven students (1.5%) (Figure 2). A small number of students, 4 (0.9%), sought advice from family members or friends, while two students (0.4%) did not take any action (Figure 2).

Our results show several noteworthy associations that might influence the use of sleep aids containing diphenhydramine hydrochloride among Jazan University students. Age did not significantly correlate with usage, as similar proportions were observed across different age groups (p=0.547) (Table 5). However, gender differences were notable, with a higher percentage of males (n=149, 60.6%) using sleep aids containing diphenhydramine hydrochloride compared to females (n=97, 50.8%) (p=0.041) (Table 5). The type of college also played a significant role, with medical students exhibiting a higher usage rate (n=141, 64.7%) compared to non-medical students (n=105, 47.9%) (p<0.001) (Table 5). Monthly income was strongly associated with OTC sleep medicine use, where students earning between 5000-10000 SAR had the highest usage rate (n=50, 81.6%), followed by those earning >10,000 SAR (n=20, 54.1%) and those earning <5000 SAR (n=186, 53.0%) (p<0.001) (Table 5). Although marital status did not show a significant association (p=0.190), married or divorced students had a higher usage rate (n=24, 66.7%) compared to single students (n=222, 55.4%) (Table 5). Employment status was significantly associated with the use of sleep aids containing diphenhydramine hydrochloride, with employed students showing a much higher usage rate (n=19, 82.6%) compared to unemployed students (n=227, 54.8%) (p=0.009) (Table 5).

**Table 5:** Association between OTC sleep medicine usage and different features (univariate analysis) The Pearson chi-square test was performed. *p-value ≤ 0.05 is considered statistically significant. n: Frequency; %: percentages; SAR: Saudi Riyal; OTC: over-the-counter

Demographic		Use of OTC Sleep Medicine (Sleep Aid Containing Diphenhydramine Hydrochloride)		p-Value
		No n (%)	Yes n (%)	
Age (years)	<18	13 (37.1)	22 (62.9)	0.547
	18-25	169 (43.9)	216 (56.1)	
	>25	9 (52.9)	8 (47.1)	
Gender	Female	94 (49.2)	97 (50.8)	0.041*
	Male	97 (39.4)	149 (60.6)	
Which college	Non-Medical	114 (52.1)	105 (47.9)	<0.001*
	Medical	77 (35.3)	141 (64.7)	
Monthly income (SAR)	<5000	165 (47.0)	186 (53.0)	<0.001*
	5000-10000	9 (18.4)	40 (81.6)	
	>10,000	17 (45.9)	20 (54.1)	
Marital status	Single	179 (44.6)	222 (55.4)	0.190
	Married/Divorced	12 (33.3)	24 (66.7)	
Employee	No	187 (45.2)	227 (54.8)	0.009*
	Yes	4 (17.4)	19 (82.6)	

Table 6 presents a multivariate analysis that reveals several significant factors influencing the usage of sleep aids containing diphenhydramine hydrochloride among Jazan University students. Notably, gender plays a significant role, with male students demonstrating a higher likelihood of using a sleep aid containing diphenhydramine hydrochloride (AOR=1.589, 95% CI: 1.059-2.383, p=0.025). Additionally, being a student in a medical college significantly increased the likelihood of using a sleep aid containing diphenhydramine hydrochloride compared to non-medical students (AOR=1.978, 95% CI: 1.333-2.936, p=0.001). Other variables, such as age, higher income, marital status, and employment, did not show statistically significant associations in this analysis. Specifically, age had an AOR of 0.600 (95% CI: 0.325-1.108, p=0.103), higher income had an AOR of 1.197 (95% CI: 0.853-1.680, p=0.299), marital status (married) had an AOR of 1.982 (95% CI: 0.896-4.385, p=0.091), and employment status had an AOR of 2.721 (95% CI: 0.861-8.604, p=0.088).

**Table 6:** Adjusted predictors of over-the-counter sleep medicine (sleep aid containing diphenhydramine hydrochloride) usage among participants (multivariate analysis) Multivariate analysis was performed using Binary logistic regression, revealing several significant factors influencing the use of sleep aids containing diphenhydramine hydrochloride among the participants. *p-value ≤ 0.05 is considered statistically significant.

Predictor	Coefficient	p-Value	Odds Ratio	95% CI	
				Lower	Upper
Age	-0.510	0.103	0.600	0.325	1.108
Gender (male)	0.463	0.025*	1.589	1.059	2.383
College (medical vs non-medical)	0.682	0.001*	1.978	1.333	2.936
Higher income	0.180	0.299	1.197	0.853	1.680
Marital status (married)	0.684	0.091	1.982	0.896	4.385
Employee (yes)	1.001	0.088	2.721	0.861	8.604
Constant	-0.345	0.659	0.709		

## Discussion

Sleep aids, categorized by their mechanisms of action, have the potential to improve sleep but can be misused, leading to adverse effects. Alhwimani et al. (2021) highlight various harmful effects resulting from the misuse of sleep medication, including dizziness or hormonal imbalance, which were the most commonly reported side effects [10]. Exercise and pharmacological interventions like antihistamines, melatonin, and benzodiazepines also help manage sleep disorders [11,12]. In Saudi Arabia, sleep disorders are common among university students [6]. Our study determined the prevalence and usage of sleep aids containing diphenhydramine hydrochloride among Jazan University students, aiming to inform better practices and highlight related risks and benefits.

Our research findings indicated that the majority of participants were 56.3% males versus 43.7% females, with most falling in the age group of 18 to 25 years (88.1%). These demographics align with university students' makeup, who are mostly male. Notably, monthly income and employment status were identified as factors influencing the use of sleep aids containing diphenhydramine hydrochloride. In particular, students with average or low incomes (5000-10,000 SAR) demonstrated higher usage rates consistent with both studies by Sosso et al. (2022) and Alhussain et al. (2023) that link lower income to sleep disorders and their OTC medication consumption [9,13]. Similarly, a study by Sosso et al. (2022) shows that people with higher incomes have a lower prevalence of sleep disorders and mood disorders, or vice versa [13]. Additionally, employed students were more inclined to use sleep aids containing diphenhydramine hydrochloride than their counterparts due to the pressures and irregular schedules associated with work, which can impact sleep patterns negatively. Our results are consistent with a previous study by Alhussain et al. (2023), which shows that OTC usage of sleep aids containing diphenhydramine hydrochloride is more prevalent among employed than non-employed individuals. Still, this relationship was not significant [9].

Moreover, a significant proportion of the participants (56.3%) had used sleep aids containing diphenhydramine hydrochloride; however, only 14% were current users. Alasmari et al. (2022) show that the most commonly used sleeping pill was the product containing diphenhydramine hydrochloride (n = 62, 73.8%) [14]. This indicates that while a sleep aid containing diphenhydramine hydrochloride is a commonly tried remedy among students, its long-term usage is less prevalent. The use of a sleep aid containing diphenhydramine hydrochloride peaked during specific times of the year for 29.1% of students, suggesting that periods of increased academic stress, such as exams, might increase its use. This pattern is consistent with studies showing that academic pressures significantly impact students' sleep quality, leading to increased reliance on sleep aids. Zunhammer et al. (2014) show that academic exam stress is known to compromise sleep quality and alter drug consumption among university students [15].

Significant gender disparities are evident in the use of OTC sleep medication, with male students demonstrating a higher likelihood of using sleep aids containing diphenhydramine hydrochloride (AOR=1.589, p=0.025). This observation contrasts with previous studies indicating a higher prevalence of sleep aid usage among females, attributed to their elevated rates of reported insomnia and sleep disturbances. Zuo et al. (2022) highlighted a higher prevalence of inadequate sleep and the use of medications commonly used for insomnia (MCUFI) in women [16]. Additionally, another study revealed that female students tend to misuse sleep aids more frequently than their male counterparts [13]. However, Aldhafiri et al. (2023) did not observe significant disparities [6]. These differences may be attributed to cultural influences and disparities in health-seeking behaviors between genders. Further in-depth research is necessary to explore these cultural influences.

It has been observed that students enrolled in medical college significantly increased the likelihood of using sleep aids containing diphenhydramine hydrochloride (AOR=1.978, p=0.001). Medical students are often under greater academic stress and have better knowledge about medications, which might contribute to higher usage rates. This finding is supported by literature indicating that medical students frequently experience high levels of stress and sleep disturbances, leading to increased use of sleep aids. Similarly, Azad et al. (2015) show that sleep disturbances are not only common among medical students, but their prevalence is also higher when compared to non-medical students and the general population [17].

Notably, 13.7% of participants reported being diagnosed with insomnia, and a substantial proportion experienced sleep disturbances due to pain (78.7% experienced it at least sometimes). This prevalence of sleep issues aligns with previous research highlighting the high incidence of sleep disturbances among university students [18]. The use of OTC sleep aids, such as the product containing diphenhydramine hydrochloride, was often driven by these disturbances, with significant proportions citing pain relief (62.4%), insomnia (55.6%), and headaches (54%) as reasons for misuse. Similarly, Alhwimani et al. (2021) show that the most common reason for using medications that contain diphenhydramine hydrochloride was insomnia (52.1%) at nighttime [10]. These findings suggest that many students may be self-medicating for medical conditions that could be solved by professional medical intervention.

To wrap up, our study on OTC sleep medication usage among Jazan University students found significant usage linked to academic stress and sleep disturbances. Contrary to some studies, we observed higher usage among males. Medical students showed greater self-medication, aligning with prior research. These findings highlight the influence of stress and accessibility on sleep aid use, underscoring the need for targeted interventions to promote safe usage and address sleep issues.

There are various implications for our research study. Initially, targeted instructional programs to enhance understanding of the risk-free use of OTC rest drugs among university students are needed. Considering the high occurrence of rest disruptions, colleges must consider supplying sources and assistance for trainees to handle stress and anxiety and boost rest health. In addition, healthcare providers must know the high prevalence of self-medication in this population and provide suitable assistance and guidance.

Limitations

This study is subject to several limitations. Firstly, it relies on self-reported data, which may introduce recall bias. Furthermore, the use of a single university sample may limit the generalizability of the findings to other populations. Additionally, the cross-sectional design of the study restrains the ability to make causal inferences. It is important to note that the study's focus on OTC sleep aids neglects the consideration of prescription medication usage and non-pharmacological sleep interventions.

## Conclusions

Our study highlights the significant use of the sleep aid containing diphenhydramine hydrochloride and other OTC sleep medications among students at Jazan University, driven by factors such as academic stress, pain, and insomnia. The findings underscore the need for better education and support for students regarding the safe use of these medications and the importance of addressing underlying sleep disturbances through professional medical intervention. Future research should explore the long-term impacts of OTC sleep medication use and develop strategies to promote healthy sleep behaviors among university students.
